# In vitro skin permeation of mitragynine: Optimisation of antioxidants for enhanced drug stability and formulation performance

**DOI:** 10.1007/s13346-025-01933-6

**Published:** 2025-08-16

**Authors:** Yee Shan Sim, Juzaili Azizi, Nelson Jeng-Yeou Chear, Siti Rafidah Yusof, Vikneswaran Murugaiyah, Su Yean Teh, Choon Fu Goh

**Affiliations:** 1 Discipline of Pharmaceutical Technology, School of Pharmaceutical Sciences, Universiti Sains Malaysia, 11800 Minden, Penang Malaysia; 2 Centre for Drug Research, Universiti Sains Malaysia, 11800 Minden, Penang Malaysia; 3 Discipline of Pharmacology, School of Pharmaceutical Sciences, Universiti Sains Malaysia, 11800 Minden, Penang Malaysia; 4 School of Mathematical Sciences, Universiti Sains Malaysia, 11800 Minden, Penang Malaysia

**Keywords:** Mitragynine, Skin permeation, Antioxidant, Drug stability, Solvent

## Abstract

The opioid-like characteristics of mitragynine with biased μ-opioid receptor activation are attractive for pain and addiction therapy due to less addictive issue. Our previous work has shown the potential of delivering mitragynine through the skin but drug degradation hindered a reliable understanding of its permeation behaviour. This study aims to optimise the use of antioxidants in both the receptor medium and formulations for in vitro permeation studies of mitragynine (5%w/v) using single solvent systems. The optimised receptor medium with 0.01%w/v of ascorbic acid in phosphate buffer saline was chosen due to a high mitragynine recovery that also allowed the detection of a higher mitragynine amount permeated. Dimethyl sulphoxide and Transcutol^®^ achieved the highest mitragynine permeation (~ 10 – 15 µg/cm^2^) and skin fluxes (~ 0.5 – 0.8 µg/cm^2^/h). While Maisine^®^ and propylene glycol achieved ~ 6 – 8 µg/cm^2^ of mitragynine permeated. Labrasol^®^ and Lauroglycol™ showed a relatively low drug permeation (~ 1 – 4 μg/cm^2^). Permeation data modelling showed that skin diffusion (high apparent diffusion coefficient) was identified as the major mechanism but skin partitioning (moderate to high apparent partition coefficient) became a determining factor for the overall permeation performance. Mass balance studies revealed low mitragynine recovery (< 80%) owing to solvent-induced degradation. Further optimisation of butylated hydroxytoluene incorporation into gel formulations with selected solvents resulted in excellent drug recovery and enhanced skin permeation, even at lower drug loadings. Overall, this study highlighted the importance of enhanced drug stability with antioxidant, facilitating a more accurate assessment of mitragynine’s skin permeation characteristics.

**Graphical Abstract:**

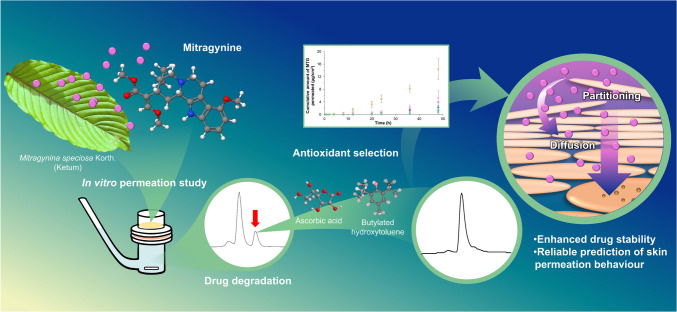

**Supplementary Information:**

The online version contains supplementary material available at 10.1007/s13346-025-01933-6.

## Introduction

*Mitragyna speciosa* Korth. is a tropical plant indigenous to Southeast Asia, particularly in parts of Malaysia and Thailand, more commonly referred to as ketum or kratom [1–3]. The ketum plant contains a number of alkaloids, with the most prevalent one being mitragynine (MTG) (~ 66% of the total alkaloid content) and other psychoactive analogues such as 7-hydroxymitragynine, speciociliatine and corynantheidine [4, 5].

Extensive studies have been conducted to elucidate the pharmacological actions of MTG, especially its stimulant-like properties and analgesic effects [6–8]. MTG is a partial μ-opioid receptor agonist and a competitive κ-opioid receptor antagonist [8]. The activation of μ-opioid receptor by MTG is biased towards G protein signalling [9]. Despite being an opioid-like compound, this biased signalling pathway makes MTG less addictive than traditional opioids [10]. Therefore, MTG has garnered considerable scientific interests as a potential treatment for chronic pain and opioid addiction [6, 11, 12].

MTG, characterised by its basic nature and poor water solubility, exhibits a low oral bioavailability (3% in rats; 69.6% in beagle dogs) due to degradation in the acidic environment of the stomach [13, 14]. To bypass the first-pass metabolism, the transdermal route for administration of MTG was first proposed by our group [15]. The evaluation of transdermal delivery of MTG is typically performed using in vitro permeation studies with Franz type diffusion cells. This experiment has been employed to evaluate the effect of single, binary and ternary solvent systems prepared using MTG and *M. speciosa* ethanolic extracts on the skin permeation [15, 16].

However, there was potential drug degradation reported which may not allow a proper understanding of the permeation behaviour of MTG [15]. Several studies have concurrently highlighted the factors inducing MTG degradation including high temperatures and extreme pH conditions [17]. Hence, the preservation of drug content in the receptor medium in Franz type diffusion cells is critical for achieving reliable quantification which is fundamental to elucidating the mechanisms of skin permeation. Equally important is maintaining drug stability within the formulation itself as this remains a key priority in ensuring effective drug delivery.

The receptor medium used in in vitro permeation tests (IVPT) must be carefully selected to closely emulate the in vivo environment, particularly to mimic the continuous clearance of drug substances by systemic circulation [18]. This can be achieved by maintaining ‘sink’ conditions throughout the experiment wherein the thermodynamic activity of the drug in the receptor medium remains < 10% of its activity in the donor compartment. In certain cases, the addition of a small amount of solubilisers such as Oleth™ 20, Volpo™ 20 or Brij™ 20 may be necessary to maintain these sink conditions without compromising membrane integrity or altering drug permeation characteristics [19–22]. Additionally, the draft guideline issued by the United States (US) Food and Drug Administration (FDA) emphasises that the receptor medium must be compatible with the skin and support both the stability and solubility of the drug [21]. Hence, we proposed the addition of an antioxidant in the receptor medium and formulations to minimise the loss of MTG during the permeation studies.

In this study, we aim to optimise the use of antioxidants in the receptor medium for in vitro permeation studies of MTG and simple gel formulations. This strategy is intended to preserve the permeated drug content, ensuring accurate quantification and a more reliable understanding of skin permeation behaviour of MTG. A total of six common solvents used including dimethyl sulphoxide (DMSO), Labrasol^®^, Lauroglycol™, Maisine^®^, propylene glycol (PG) and Transcutol^®^ which have previously employed in several studies for topical and transdermal formulation designs [23–27]. The selected solvents were used for optimisation of antioxidants before incorporation into a gel system for skin permeation evaluation. With the use of mathematical modelling, this study provides further fundamental insights into the skin permeation behaviour of MTG through the analysis of permeation parameters.

## Materials and method

### Materials

MTG standard (≥ 95% purity) was obtained from the Centre for Drug Research, Universiti Sains Malaysia. High performance liquid chromatography (HPLC) grade acetonitrile and methanol and sodium hydroxide pellets were purchased from Fisher Scientific (Waltham, MA, US). Formic acid (85%) and hydrochloric acid (37%) were obtained from Quality Reagent Chemical (QRëCTM, New Zealand). Hydrogen peroxide (30%) solution was acquired from R&M Chemicals (Selangor, Malaysia). DMSO, dithiothreitol (DTT), hydroxypropyl methylcellulose (HPMC) and sodium azide were purchased from Sigma Aldrich (St. Louis, MO, US). Glycerol and PG were obtained from JT Baker (Phillipsburg, NJ, US) while Labrasol^®^, Lauroglycol™, Maisine^®^ and Transcutol^®^ were generous gifts from Gattefossé (Saint-Priest, France). Phosphate buffer saline (PBS) solution (pH 7.3 ± 0.2 at 25 °C) was prepared by dissolving one Dulbecco A tablet purchased from Oxoid Ltd. (Basingstoke, United Kingdom) in 100 mL of distilled water. Ascorbic acid, butylated hydroxytoluene (BHT) and methylparaben were purchased from Ungerer Australia (NSW, Australia). Porcine ear skin was obtained from a local abattoir in Batu Maung, Penang, Malaysia. The fat layer was removed and the hair was trimmed before storing at − 20 °C when not in use.

### Characterisation of MTG

The thermal profile of MTG was obtained using PerkinElmer^®^ Pyris 6 differential scanning calorimetry (DSC) (Waltham, MA, US). Approximately 2–5 mg of MTG was weighed and placed in a standard aluminium pan (PerkinElmer^®^, Waltham, MA, US) before heating from 30–300 °C at 10 °C/min under a dry nitrogen gas flow of 50 mL/min. The data were analysed using Pyris Data Analysis software (Waltham, MA, US).

Attenuated total reflectance-Fourier transform infrared (ATR-FTIR) spectra of MTG was obtained using a FTIR spectrophotometer (Shimadzu Prestige 21 spectrometer, Shimadzu Corporation, Kyoto, Japan) coupled with an ATR diamond crystal over a wavenumber range of 4000–600 cm^−1^. A total of 32 scans was taken with a resolution of 4 cm^−1^ and the spectra were analysed using OriginPro^®^ software (OriginPro^®^ 8.5, OriginLab Corporation, Northampton, MA, US).

X-ray diffraction (XRD) analysis was conducted using Bruker D8 Advance XRD (Bruker, Billerica, MA, US). The MTG sample was exposed to an X-ray radiation source produced by a copper anode (Kα = 1.54Ǻ) with a step size of 0.02° over the 2*θ* range of 5° – 80° at 40 kV and 40 mA. The XRD data were analysed using OriginPro^®^ software aforementioned.

### Forced degradation studies of MTG

Forced degradation studies of MTG was conducted in accordance with the International Council on Harmonisation (ICH) guideline in extreme environments (acidic, alkali, heat, oxidation and ultraviolet (UV) light exposure) [28, 29]. MTG solutions (1 mg/mL) in methanol was prepared with the content of 0.1 M hydrochloric acid (acid-induced degradation), 0.1 M sodium hydroxide (alkaline degradation) and 30%v/v hydrogen peroxide (oxidation) before storing at 60 °C over 24 h. The effect of temperature (heat) on MTG was investigated by heating a known amount of MTG powder at 100 °C over 24 h. The effect of UV light exposure was conducted by exposing a known amount of MTG powder to UV light in a UV steriliser (UV wavelength: 254 nm) (Boboduck^®^, Shanghai, China) over 24 h. Samples were taken at 6, 12 and 24 h for dissolution and/or dilution before HPLC analysis (Sect. HPLC analysis).

### Antioxidant selection for receptor medium

The selection of antioxidant for receptor medium was conducted by adding 0.01, 0.05 and 0.1%w/v of DTT and ascorbic acid in MTG solution (50 μg/mL) prepared using PBS at 32 ± 1 °C over 96 h. The drug content was analysed using HPLC (Sect. HPLC analysis) at 24, 48, 72 and 96 h.

### In vitro permeation and mass balance studies

The in vitro permeation study was performed in triplicate using Franz diffusion cell apparatus with a diffusion area of ~ 0.8 cm^2^ and a receptor volume of ~ 3 mL. The porcine skin was thawed at room temperature and cut into an appropriate size before mounting on the Franz diffusion cells. The receptor compartment was filled with PBS containing 0.02%w/v of sodium azide and a selected antioxidant. The Franz diffusion cells were placed into a water bath to maintain the skin temperature at 32 ± 0.5 °C. An infinite dose (40 μL) of 5%w/v of MTG dissolved in simple solvent systems as mentioned in Sect. Stability studies of MTG in simple solvent systems was loaded on the skin and occluded with Parafilm^®^ M. An aliquot of 200 μL was collected from the receptor compartment at predetermined time points (0.5, 1, 2, 4, 8, 12, 20, 24, 36 and 48 h) and was replaced with the same aliquot of fresh PBS to maintain a 'sink' condition. After 48-h permeation studies, the mass balance studies were performed. The samples were extracted for MTG content overnight before HPLC analysis.

In order to validate the surface wiping procedure for mass balance studies, MTG dissolved in the selected solvent (PG) was loaded in the same manner as described previously but without the addition of permeation medium in the receptor compartment [30]. Immediately post-application, any remaining MTG was swabbed from the skin surface with cotton buds and added into an Eppendorf tube. Subsequently, the surface of the skin was washed twice with 500 μL of methanol before adding to the same Eppendorf tube containing the cotton buds. The skin was removed from the dissembled Franz diffusion cell and cut into pieces before placing in another Eppendorf tube containing 1 mL of methanol. All samples were vortexed and analysed using HPLC (Sect. HPLC analysis). The validation showed that the skin surface washes demonstrated an excellent percentage recovery of MTG (100.97 ± 0.07%, *n = *3) with no detection of MTG in the skin.

### HPLC analysis

The HPLC analysis of MTG was performed using a validated method previously reported [15]. Samples were analysed using Shimadzu liquid chromatographic system (Kyoto, Japan) with SPD-20A UV–vis detector set at 254 nm. A reverse phase ZORBAX Eclipse Plus C_18_ column (Agilent Technologies, 150 × 4.6 mm ID × 3.5 μm) coupled with a C_18_ guard column (Phenomenex SecurityGuard™, 4 × 3.0 mm ID × 5 μm) was employed as the stationary phase. A gradient elution method with acetonitrile and 0.1%v/v of formic acid as the mobile phase (30:70 to 70:30 (v/v) over 1 min and held until 4 min; then returned to 30:70 (v/v) from 4 to 6 min and held up until 10 min) at a flow rate of 1.2 mL/min was used. The injection volume was set at 100 μL and the total run time was 10 min with a retention time of ~ 4 min. The limits of detection and quantification were 0.47 and 1.43 μg/mL for a lower concentration drug range (0.5–10 μg/mL) and 4.29 and 13.01 μg/mL for a higher concentration drug range (10–175 μg/mL), respectively. The linearity for both calibration curves was excellent (*R*^*2*^ > 0.999).

### Analysis of in vitro permeation data using mathematical model

To estimate the permeation parameters (*P*_*1*_ and *P*_*2*_), the in vitro permeation data were fitted to the infinite dose model (Eq. 1) using non-linear least squares [31]. This infinite dose model was obtained by solving the diffusion equation using Laplace transform, which was subsequently numerically inverted using Weeks method and fitted to in Python 3.13.3 (Python Software Foundation, Wilmington, DE, US).

1 $$\overline{Q }\left(s\right)=J\cdot \frac{A}{s}=\frac{{P}_{1}A{C}_{d}}{s\sqrt{\frac{s}{{P}_{2}}}sinh\left(\sqrt{\frac{s}{{P}_{2}}}\right)}$$

Here, $\overline{Q }\left(s\right)$ is the cumulative amount of drug permeated, *J* is the permeation flux, *A* is the permeation area, *C*_*d*_ is the initial concentration, *h* is the membrane thickness and *s* is the Laplace variable. The apparent partition and apparent diffusion coefficients, *P*_*1*_ and *P*_*2*_, are related to the *h* and the diffusion (*D*) and partition (*K*) coefficients, as defined in Eq. 2 and Eq. 3 [32]. The goodness of fit was measured based on the coefficient of determination (*R*^*2*^) and root mean square error (RMSE).

2 $${P}_{1}=Kh$$

3 $${P}_{2}=\frac{D}{{h}^{2}}$$

The permeability coefficient (*K*_*p*_), steady-state flux (*J*_*ss*_) and lag time (*t*_*lag*_) can then be calculated using Eqs. 4, 5, and 6 [33] with the estimated values of *P*_*1*_ and *P*_*2*_ from the curve fitting.

4 $${K}_{p}={P}_{1}{P}_{2}$$

5 $${J}_{ss}={K}_{p}{C}_{d}$$

6 $${t}_{lag}=\frac{{h}^{2}}{6D}$$

Other permeation parameters were determined experimentally, where *A* was 0.7–0.8 cm^2^, *h* was 0.0586 cm, *C*_*d*_ was 50 mg/mL and the volume of drug loaded was 40 µL.

### Stability studies of MTG in simple solvent systems

The stability of MTG (200 μg/mL) in the solvents used including DMSO, Labrasol^®^, Lauroglycol™, Maisine^®^, PG and Transcutol^®^ with and without BHT at 32 ± 1 °C was investigated every 24 h for 96 h by analysing drug content (diluted suitably) using HPLC (Sect. HPLC analysis).

### Preparation and evaluation of skin permeation of gel formulations containing MTG

A hydrophilic gel base was formulated by dispersing 10%w/v of HPMC in hot distilled water (~ 80 °C) until completely solubilised before adding 1% of MTG, 10%w/w of glycerol (as emollient), 0.1%w/v of methylparaben and 10%w/v of selected solvents. BHT at the optimised concentration was also added accordingly. The gels were evaluated for their drug content by mixing the gel in methanol for overnight extraction of MTG before HPLC analysis (Sect. HPLC analysis). In vitro drug release studies were carried out with a similar experimental setup as described in Sect. In vitro permeation and mass balance studies but porcine skin was replaced with a 0.45 µm nylon membrane filter and 40 μL of gel was loaded on the membrane at the donor compartment. Subsequent in vitro permeation and mass balance studies were performed as described in Sect. In vitro permeation and mass balance studies by loading 40 μL of the gel on the skin.

### Statistical analysis

Statistical analysis was conducted using IBM^®^ SPSS^®^ version 25 (IBM Corp., Armonk, NY, US). The data were evaluated using one-way ANOVA with post hoc Tukey’s test. A *p* value of < 0.05 was considered as the minimum level of significance in all cases. All results were expressed as mean ± standard deviation (SD).

## Results and discussion

### Characterisation of MTG

**Fig. 1:**
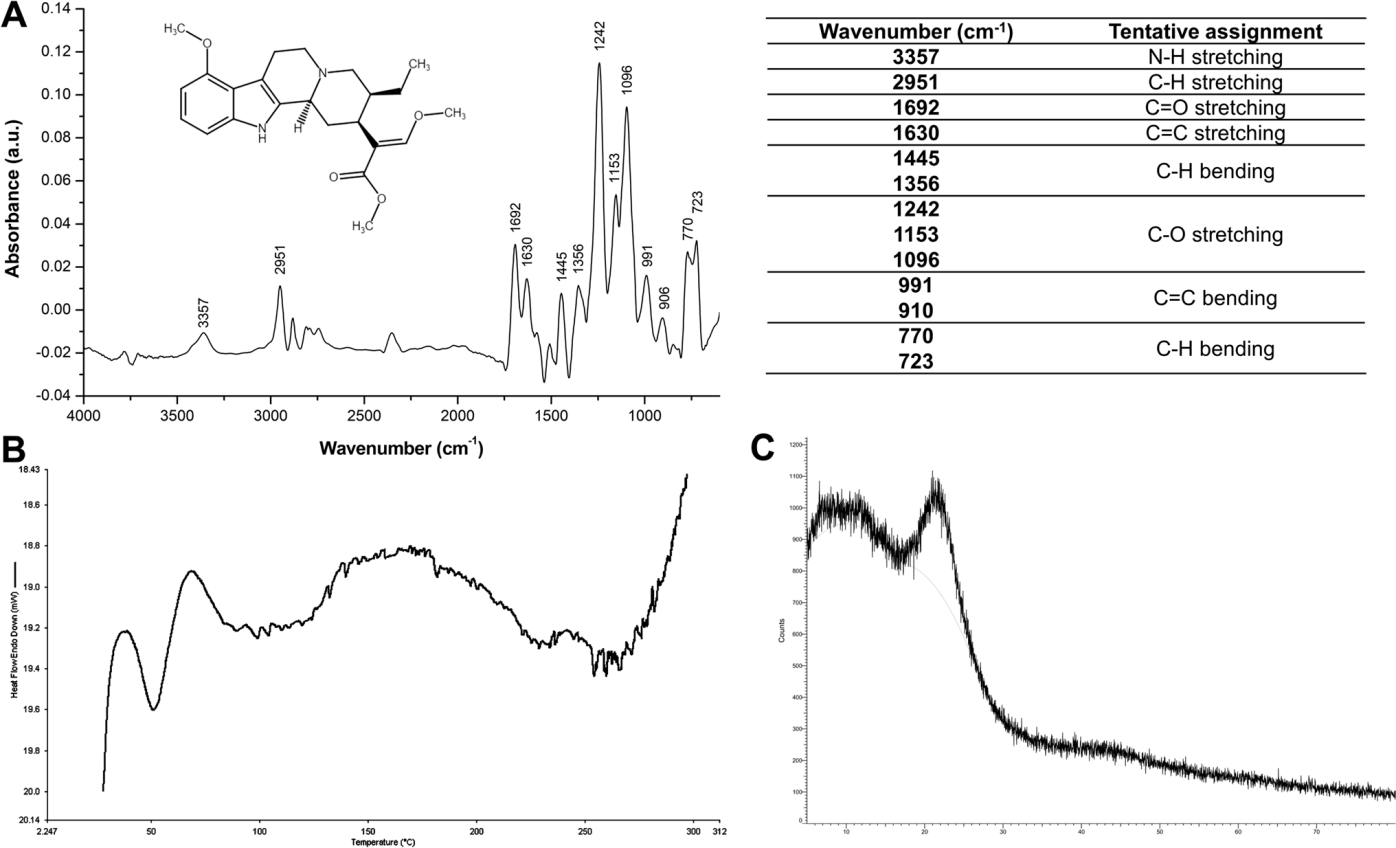
(**A**) ATR-FTIR spectrum with its respective spectral assignments, (**B**) DSC thermogram and (**C**) XRD profile of MTG

MTG was characterised for its physicochemical properties as shown in Fig. 1. The FTIR analysis of MTG showed prominent peaks corresponding to N–H stretching (3357 cm^−1^), C = C stretching (1630 cm^−1^), C-H stretching/bending (2951, 1445, 1356, 770 and 723 cm^−1^) and C-O stretching (1242, 1153 and 1096 cm^−1^) (Fig. 1A). Due to the limited reports, MTG can be identified and compared to structurally related compounds containing indole groups such as harmine. According to Prasad Kushwaha, Baidya and Patil [34], FTIR analysis of harmine revealed five characteristic peaks with corresponding spectral assignments at 3147 cm^−1^ (N–H stretching), 1617 cm^−1^ and 1599 cm^−1^ (aromatic C = C stretching), 1453 cm^−1^ (C-H alkane bending) and 1026 cm^−1^ (C-O stretching). Moreover, Bei, Zhou, You, Yuan, Chen, Xia, Liu, Jin, Hu, Zhu, Zhang, Zhang and Zhang [35] reported two peaks for harmine at 1636 cm^−1^ and 816 cm^−1^, likely attributed to the aromatic stretching of the C = C and C-H from the benzene ring of the indole group. Overall, the structure of MTG was confirmed via FTIR chemical identification.

Three major peaks were observed in the DSC thermogram of MTG (Fig. 1B). A sharp peak at 50 °C has not been reported but this could be attributed to trace impurities present in the MTG sample with ≥ 95% purity [36]. The broad peak around 100 °C is commonly associated with moisture evaporation while the second broad and noisy peak near 250 °C suggests possible thermal decomposition of MTG. The analysis of MTG using DSC has been scarcely reported in the literature. Beng, Hamdan, Siddiqui, Mordi and Mansor [37] reported a melting point of approximately 103–105 °C but the thermogram was not provided. This renders the findings inconclusive because no distinct endothermic peak was observed within this range in our current work. Therefore, the current DSC results primarily suggest the absence of crystallinity in MTG. The XRD profile of MTG (Fig. 1C) exhibits a halo pattern, lacking the sharp diffraction peaks typically observed in crystalline substances. Together, the DSC and XRD results indicate that MTG likely exists in an amorphous form.

### Forced degradation studies of MTG

Table 1 presents the percentage recovery of MTG under all stressed conditions over 24 h. The results indicate that MTG underwent the most significant degradation under the oxidative and alkaline conditions (~ 95%) and this was followed by thermal degradation (~ 90%). In contrast, degradation under acidic conditions and UV exposure was comparatively lower (40–60%) although still considered substantial. The instability of MTG under acidic and alkaline media at elevated temperatures is consistent with findings reported in the previous studies [17, 38]. Ramanathan, Parthasarathy, Murugaiyah, Magosso, Tan and Mansor [38] proposed drug degradation after observing ~ 11.2% of MTG loss at pH 4 over 24 h. Later, MTG loss up to ~ 20% was also reported in basic environments (pH 8 and 10) and at 40 °C over 8 h [17].

**Table 1:** Percentage recovery of MTG in forced degradation studies (*n* = 3, mean ± SD)

Conditions	Percentage recovery of MTG (%)		
Time (h)	6	12	24
Acid hydrolysis (0.1 M hydrochloric acid)	70.66 ± 2.95	67.67 ± 3.14	58.09 ± 2.03
Base hydrolysis (0.1 M sodium hydroxide)	17.60 ± 0.43	7.05 ± 0.47	5.33 ± 0.56
Oxidation (30%v/v hydrogen peroxide)	8.33 ± 0.63	7.38 ± 0.62	5.23 ± 0.62
Thermal degradation (100 °C)	56.03 ± 3.68	35.08 ± 0.60	9.63 ± 0.16
Photolytic degradation (UV exposure at 254 nm)	68.41 ± 3.55	69.99 ± 0.61	63.01 ± 4.59

In the in vitro permeation studies, MTG is potentially susceptible to degradation, likely due to exposure to heat, light, and ambient conditions. In addition, MTG was likely in the amorphous state as evaluated in Sect. Characterisation of MTG which may have made it more susceptible to chemical degradation. The use of a slightly elevated temperature (~ 32 °C) to simulate human skin surface temperature during the experiment is unavoidable but it raises concerns about oxidative degradation. This highlights the importance of incorporating an antioxidant into the receptor medium and potentially into the formulation itself to prevent drug loss and ensure accurate quantification of MTG permeation.

### Antioxidant selection for receptor medium

**Fig. 2:**
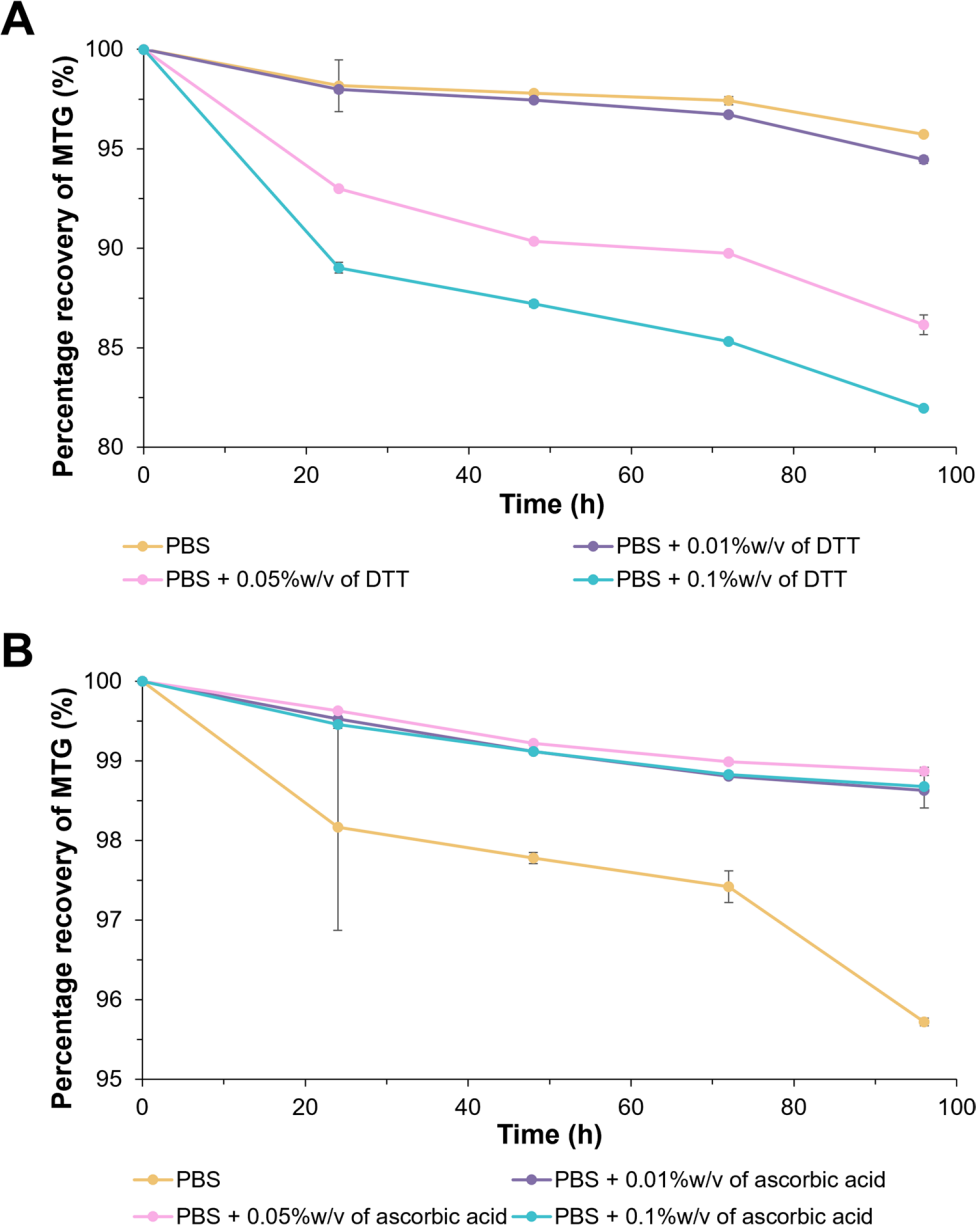
Percentage recovery of MTG in PBS with 0.01, 0.05 and 0.1%w/v of (**A**) DTT and (**B**) ascorbic acid over 96 h at 32 °C (*n = *3, mean ± SD)

The stability profile of 50 µg/mL of MTG solution in PBS with the addition of antioxidants was shown in Fig. 2. Without the use of an antioxidant, there was a slight decrease in the percentage recovery of MTG to ~ 96% after 96 h. However, there was a marked decrease in the MTG recovery when DTT was used as low as ~ 81% and the drug loss was more substantial at a higher DTT content. On the other hand, ascorbic acid was shown to minimise the loss of MTG and the percentage recovery of MTG remained almost similar (> 99%) regardless of the antioxidant concentration.

The degradation of MTG has been previously reported to be induced by extreme pH and temperatures as discussed previously [17, 38]. Our forced degradation studies also identified oxidation as the primary degradation pathway, with only ~ 8% of drug recovery after 6 h (Sect. Forced degradation studies of MTG), significantly lower than recoveries observed under other stress conditions such as acid and alkali hydrolysis, thermal degradation and photolysis. Moreover, the experimental setup for standard in vitro permeation studies does not typically expose samples to extreme pH values or elevated temperatures. Based on these observations, the present study introduced the addition of an antioxidant as an approach to maintain drug stability and ensure accurate drug quantification during in vitro permeation experiments.

Recent US FDA draft guideline related to the use of IVPT [21] recommends avoiding the use of organic solvents and alcohols in the receptor medium as their presence may compromise the validity of the experiments. Despite, the use of alcohols such as ethanol and isopropanol as well as non-ionic surfactants such as polyethylene glycol-20-oleyl ether (Brij™ O20, Volpo™ 20) has been previously reported mainly for maintaining 'sink' conditions [18, 39, 40]. The use of preservatives such as sodium azide [41–43] usually at low concentrations (0.005–1%), which do not affect skin integrity, may be considered appropriate.

DTT and ascorbic acid are common antioxidants for pharmaceutical applications. DTT is a typical reducing agent used to stabilise proteins with disulphide bonds or thiol groups [44] while ascorbic acid can be found in skincare and pharmaceutical products [45]. DTT has been previously used to preserve drug stability in the in vitro permeation experiments at 0.05%w/v [46]. However, it is noteworthy that DTT unexpectedly caused drug loss in the present study. Both antioxidants have previously been reported to enhance the permeation of various compounds, including sodium diclofenac, mannitol, and sucrose, across rat and human skin [47, 48]. Although such an effect is undesirable, the current study utilised a very low concentration of antioxidants, substantially lower than those reported in earlier studies (as low as 0.19%w/v of DTT and 0.44%w/v of ascorbate) which is unlikely to compromise the skin integrity. Hence, 0.01%w/v of ascorbic acid was selected as the antioxidant of choice for the following in vitro permeation studies.

### In vitro permeation and mass balance studies

**Fig. 3:**
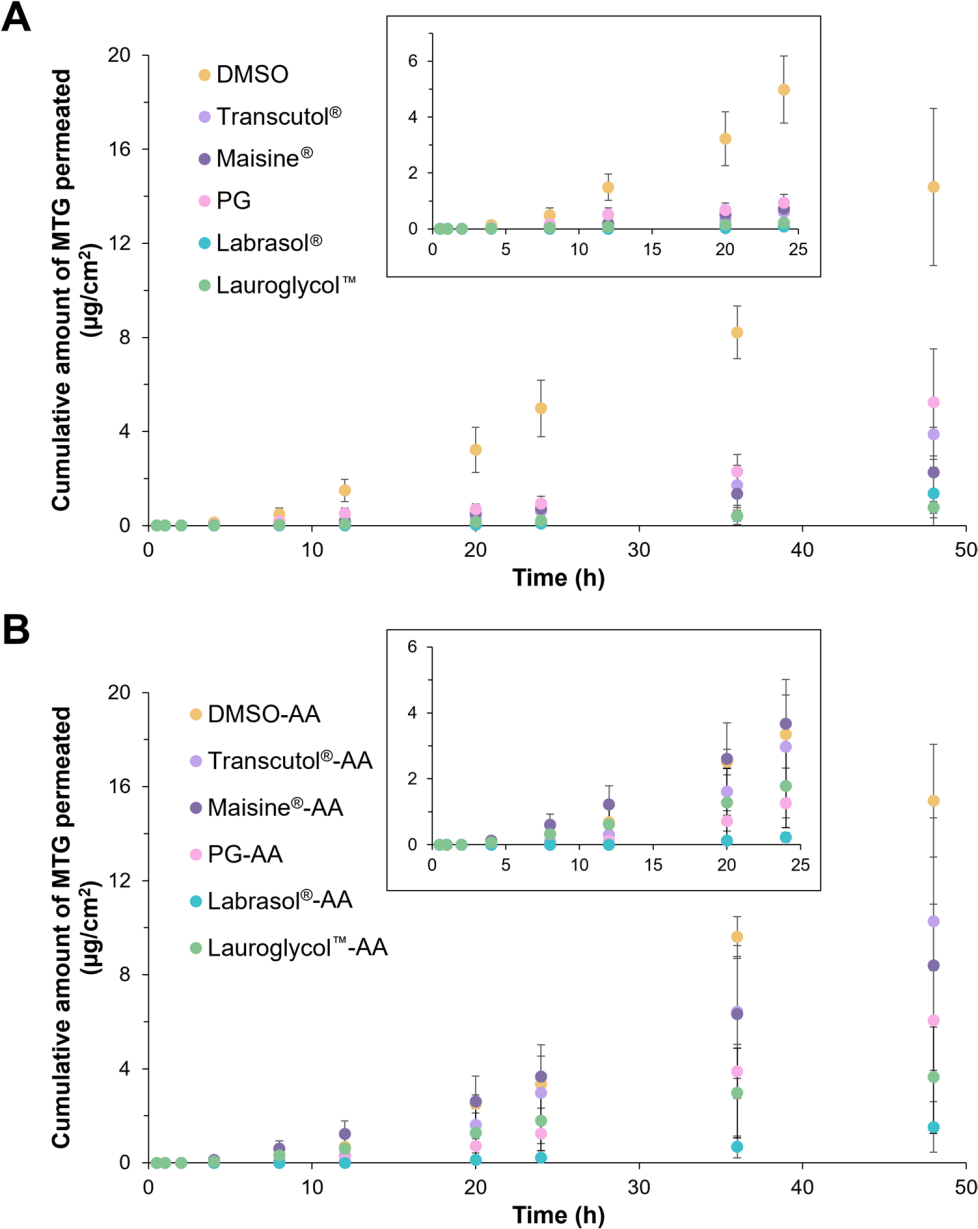
In vitro skin permeation profiles of 5%w/v of MTG in various solvents (**A**) without and (**B**) with the addition of ascorbic acid in the receptor medium over 48 h (*n = *3, mean ± SD; inset shows profile up to 24 h)

Figure 3 illustrates the in vitro permeation profiles of MTG in solvents using different receptor media over 48 h. The sample name with ‘-AA’ denoted the addition of 0.01%w/v of ascorbic acid to the PBS. The presence of ascorbic acid in the receptor medium generally showed a higher cumulative amount of MTG permeated as compared to the corresponded solvent system due to the preservation of the stability of MTG. The individual solvent comparisons can be found in Supplementary Material.

Without the presence of ascorbic acid (Fig. 3A), DMSO showed the highest skin permeation of MTG among all solvents, achieving 14.40 ± 3.34 μg/cm^2^ of MTG permeated with a high skin flux of 0.52 ± 0.15 μg/cm^2^/h. This distinctly differentiates DMSO from the other solvents, indicating its strong potential as a skin penetration enhancer. While the rest of the solvents only showed MTG permeation after 12 h with a cumulative amount of MTG permeated of < 4 μg/cm^2^ (Transcutol^®^: 3.89 ± 1.41 μg/cm^2^; Maisine^®^: 2.25 ± 1.93 μg/cm^2^; PG: 5.24 ± 2.28 μg/cm^2^; Lauroglycol™: 0.77 ± 0.25 μg/cm^2^; Labrasol^®^: 1.36 ± 1.45 μg/cm^2^).

The addition of antioxidant exhibited distinctive differences for most solvents except Labrasol^®^ which showed minimal change (Labrasol^®^-AA: 1.53 ± 1.08 μg/cm^2^; Labrasol^®^: 1.36 ± 1.45 μg/cm^2^), implying its limited enhancement effect. Among all tested solvents, DMSO-AA consistently exhibited the highest cumulative amount of MTG permeated (15.40 ± 2.40 μg/cm^2^) and steady-state flux (0.77 ± 0.18 µg/cm^2^/h), reaffirming its superior skin penetration enhancement effect.

Following this, Transcutol^®^-AA and Maisine^®^-AA shared similar skin permeation profiles of MTG with the exception of a higher cumulative permeated amount of MTG using Transcutol^®^-AA (10.27 ± 4.40 μg/cm^2^) than Maisine^®^-AA (8.39 ± 2.61 μg/cm^2^). The MTG amount permeated was 3–4 times higher than those without the use of ascorbic acid, signifying the role of antioxidant in preserving the drug for an appropriate evaluation of its skin permeation potential. Despite PG-AA demonstrating a higher MTG permeation, the improvement was not substantial (PG-AA: 6.06 ± 2.12 μg/cm^2^; PG: 5.24 ± 2.28 μg/cm^2^). Lauroglycol™ showed a marked change in the presence of ascorbic acid with ~ fivefold higher MTG permeated (Lauroglycol™-AA: 3.65 ± 2.40 μg/cm^2^; Lauroglycol™: 0.77 ± 0.25 μg/cm^2^).

A recent in vitro permeation study by Tuntiyasawasdikul, Junlatat, Tabboon, Limpongsa and Jaipakdee [16] using 2%w/v of *M. speciosa* ethanolic extract (containing ~ 0.2% of MTG) in PG and Transcutol^®^ (diffusion area: ~ 2.3 cm^2^; dosing volume: 500 μL) achieved a cumulative amount of MTG permeated after 24 h of ~ 50 μg/cm^2^ which was tenfold higher than our work. The MTG dose was also lower (~ 0.5 mg/cm^2^) than the current work (2.5 mg/cm^2^). The enhanced MTG permeation in their study is likely attributed to the inclusion of 2%w/v Tween^®^ 80 in the receptor medium, which may have facilitated back-diffusion into the skin, promoting further drug permeation. The addition of a surfactant to enhance drug permeation was not considered here as the saturated solubility of MTG in PBS (77.10 ± 5.17 μg/mL at 32 °C) was much higher than the recommended concentration limit of ~ 10 μg/mL where a solubiliser is required [15, 40].

Table 2 shows the skin permeation parameters of MTG (*P*_*1*_,* P*_*2*_, *K*_*p*_, *J*_*ss*_ and *t*_*lag*_) fitted using an infinite dose model. Due to the limited time points for an appropriate fitting, Labrasol^®^ and Lauroglycol™ were not considered for the evaluations. PG and Transcutol^®^ achieved high *P*_*1*_, indicating enhanced drug partitioning into the skin. While DMSO and Maisine^®^ showed a higher *P*_*2*_ which suggested drug diffusion as the main drug permeation mechanism. However, the differences for both *P*_*1*_ and *P*_*2*_ results were not significant due to a huge variation (one-way ANOVA, *p* > 0.05). The well-balanced contributions of both *P*_*1*_ and *P*_*2*_ by DMSO resulted in the highest *K*_*p*_ but this showed no significant difference with PG and Transcutol^®^ (*p* > 0.05). Nevertheless, this also made DMSO achieving the shortest *t*_*lag*_.

**Table 2:** Skin permeation parameters fitted using an infinite dose model for permeation profiles of 5%w/v of MTG in various solvents (*n = *3, mean ± SD; one-way ANOVA with post-hoc Tukey’s test)

Solvent	*P*_*1*_ (× 10^–4^ cm)	*P*_*2*_ (× 10^–3^ h^−1^)	*K*_*p*_ (× 10^–6^ cm/h)	*J*_*ss*_ (µg/cm^2^/h)	*t*_*lag*_ (h)
DMSO	8.90 ± 5.52	13.40 ± 4.73	10.46 ± 3.03	0.52 ± 0.15	13.52 ± 4.70
PG	25.55 ± 25.22	7.33 ± 6.13	9.59 ± 7.34	0.48 ± 0.37	33.85 ± 20.70
Transcutol^®^	26.94 ± 23.48	4.29 ± 1.70	8.89 ± 3.69	0.44 ± 0.18	44.70 ± 22.17
Maisine^®^	1.89 ± 1.95	12.86 ± 5.15	2.09 ± 1.81	0.10 ± 0.09	14.19 ± 4.62
DMSO-AA	20.35 ± 8.07	8.01 ± 1.91	15.30 ± 3.61	0.77 ± 0.18^d^	21.53 ± 4.53
PG-AA	9.49 ± 3.95	7.17 ± 0.89	6.59 ± 2.31^c^	0.33 ± 0.12	23.50 ± 2.93
Transcutol^®^-AA	10.40 ± 4.15	8.76 ± 0.55	9.26 ± 4.32	0.46 ± 0.22	19.07 ± 1.17
Maisine^®^-AA	2.18 ± 0.48^a^	25.90 ± 8.44	5.42 ± 1.35^c^	0.27 ± 0.07	7.05 ± 2.82^e^
Labrasol^®^-AA	6.83 ± 5.64^a^	4.33 ± 0.52	2.79 ± 2.13^c^	0.14 ± 0.11	38.85 ± 4.39^e,f^
Lauroglycol™-AA	0.65 ± 0.09^a^	33.50 ± 18.62^b^	2.27 ± 1.37^c^	0.11 ± 0.07	6.39 ± 4.02^e^

^a^ Significantly different from DMSO-AA

^b^ Significantly different from other -AA solvents

^c^ Significantly different from DMSO-AA

^d^ Significantly different from other -AA solvents

^e^ Significantly different from DMSO-AA, PG-AA and Transcutol^®^-AA

^f^ Significantly different from Maisine^®^-AA and Lauroglycol™-AA

With the incorporation of ascorbic acid in the receptor medium, the overall permeation pattern remained largely unchanged, with DMSO-AA outperforming nearly all permeation parameters except *P*_*2*_ (*p* < 0.05). Despite having a higher *P*_*2*_, Lauroglycol™-AA and Maisine^®^-AA still suffered from fairly low *J*_*ss*_ due to a very low skin partitioning (*P*_*1*_). Regardless, a higher skin diffusion could benefit a lower *t*_*lag*_.

Table 3 shows the results of mass balance studies following the previous in vitro skin permeation studies. Similar to the skin permeation findings, the MTG content in the skin was generally found to be higher with the presence of ascorbic acid. Nevertheless, the overall percentage recovery fell below the acceptable percentage recovery range (80–120%) set by the Organisation for Economic Co-operation and Development (OECD), suggesting a possibility of loss due to drug degradation [20]. This occurred to the MTG left on the skin, indicating potential drug degradation at the donor chamber during the permeation studies. This was supported by the stability results of MTG in the solvent systems, where the percentage recovery of MTG was < 80% in all solvents after 96 h (Sect. Stability studies of MTG in simple solvent systems). There was only a weak linear correlation (*R*^*2*^ = 0.4) between the percentage recovery of MTG and the total drug recovery in the mass balance results (without ascorbic acid). Surprisingly, a strong negative correlation was observed (*R*^*2*^ = −0.9) when ascorbic acid was added to the receptor medium. As the exact mechanism of MTG degradation in solvents remains unknown, further studies are warranted to understand the interactions between MTG and the solvents which could be beneficial for future MTG formulation designs.

**Table 3:** Mass balance results after skin permeation studies of 5%w/v of MTG in different solvents (*n = *3, mean ± SD)

Solvent	Permeation		Skin extraction (%)	Washing (on skin surface) (%)	Total percentage (%)
	(µg/cm^2^)	(%)			
Transcutol^®^	3.89 ± 1.41	0.19 ± 0.07	2.08 ± 1.11	71.94 ± 8.01	75.39 ± 9.19
Maisine^®^	2.26 ± 1.93	0.11 ± 0.10	0.45 ± 0.18	61.67 ± 5.42	62.51 ± 5.69
Labrasol^®^	1.36 ± 1.45	0.07 ± 0.07	3.67 ± 0.45	65.82 ± 5.20	70.07 ± 5.72
Lauroglycol™	0.77 ± 0.25	0.04 ± 0.01	0.52 ± 0.38	66.42 ± 2.26	67.37 ± 2.65
DMSO-AA	15.40 ± 2.40	0.77 ± 0.12	0.91 ± 0.19	48.69 ± 0.86	50.68 ± 1.17
PG-AA	6.06 ± 2.13	0.30 ± 0.11	1.04 ± 0.20	45.95 ± 5.07	47.60 ± 5.37
Transcutol^®^-AA	10.27 ± 4.40	0.51 ± 0.22	2.29 ± 0.57	50.98 ± 9.49	54.57 ± 10.28
Maisine^®^-AA	8.39 ± 2.61	0.42 ± 0.13	1.34 ± 0.55	60.12 ± 1.46	62.55 ± 2.14
Labrasol^®^-AA	1.53 ± 1.08	0.08 ± 0.05	3.23 ± 1.80	70.50 ± 5.24	75.65 ± 7.09
Lauroglycol™-AA	3.65 ± 2.40	0.18 ± 0.12	1.96 ± 1.22	57.28 ± 15.05	59.42 ± 16.39

### Influence of solvents on the skin permeation enhancement for MTG

In the skin permeation investigations, DMSO, PG and Transcutol^®^ were found to be excellent solvents in enhancing MTG permeation. These solvents have also been previously employed in pharmaceutical products as drug solubilisers apart from serving as skin penetration enhancers [27]. The discussion herein focuses on the results obtained from the experiments added with antioxidants due to the interests on the reliable evaluation.

The exceptional skin permeation enhancement by DMSO is closely related to its strong solvency effect due to its polar aprotic behaviour which may form a drug reservoir in the skin [49–53]. At low concentrations of < 20%, DMSO can interact with the stratum corneum (SC) protein and the influence enhances at > 40% which can lead to the protein denaturing and SC lipid modification [54–57]. This may contribute to the highest skin partitioning (*P*_*1*_) with a moderately high skin drug diffusion (*P*_*2*_) in the current work under the influence of DMSO.

On the other hand, Transcutol^®^ is a specially designed solvent for skin permeation enhancement and possesses exceptional solvency power for a wide range of drugs. The permeation enhancement benefits of Transcutol^®^ are mainly attributed to its ability of partitioning into the skin to create a temporary intracutaneous drug depot [58–61]. This observation was supported by a high *P*_*1*_ (Table 2) among all solvents except DMSO and a higher percentage of MTG recovered from the skin in mass balance studies (Table 3). Recent works on niacinamide [62] and methadone [63] have also shown the remarkable improvement of skin permeation by Transcutol^®^ even comparing to glycols (PG, PG monolaurate and tri-PG).

PG is one of the most common solvents used due to its high drug solubilising ability and safety profile [64]. In the current study, PG documented a moderate amount of MTG permeated similar to Transcutol^®^. PG’s ability to cause the SC lipid disordering and keratin protein denaturation as well as dehydration has been well documented for skin permeation enhancement [65–68]. Depletion of PG from the donor chamber due to solvent evaporation and its fast diffusion behaviour is also the main reason for a higher drug diffusion (*P*_*2*_) [69, 70]. This could explain the contribution of PG in enhancing the skin permeation of MTG.

The rest of the solvents including Maisine^®^, Lauroglycol™ and Labrasol^®^ exhibited lower enhancement of MTG permeation compared to the previously discussed solvents. Maisine^®^ is more commonly used as an oily phase/vehicle in emulsions and liposomes [71, 72]. Although its permeation enhancement effect is unclear, Maisine^®^ is expected to modify the lipid configurations of the SC, similar to other fatty acid esters, due to its composition of esterified glycerides with oleic acid and linoleic acid, thereby improving the skin penetration of lipophilic compounds such as MTG [38, 73]. This is also reflected in a higher *P*_*2*_ value than *P*_*1*_ value using Maisine^®^.

Both surfactants, Labrasol^®^ and Lauroglycol™ contributed to a very minimal skin permeation enhancement for MTG. Previous work by Bonina, Carelli, Di Colo, Montenegro and Nannipieri [74] has also reported a low caffeine and testosterone permeation for Labrasol^®^ as compared to solvents such as Labrafil^®^, PG-dipelargonate and Transcutol^®^ which may be due to a lower human callus/vehicle partition coefficient. While, the use of Lauroglycol™ as a skin penetration enhancer in simple, binary and ternary solvent systems has been reported for several studies [65–67]. Despite limited literature available, Irion, Garrison and Abraham [75] suggested that Lauroglycol™ partitioned into the SC to perturb the SC lipid bilayers for an enhanced estradiol permeation. The current work showed that Labrasol^®^ and Lauroglycol™ achieved a higher *P*_*2*_ than *P*_*1*_, indicating the important role of MTG diffusion rather than partitioning by these solvents. Nevertheless, the fitting for both surfactants may be constrained by the low level of MTG permeation.

### Stability studies of MTG in simple solvent systems

**Fig. 4:**
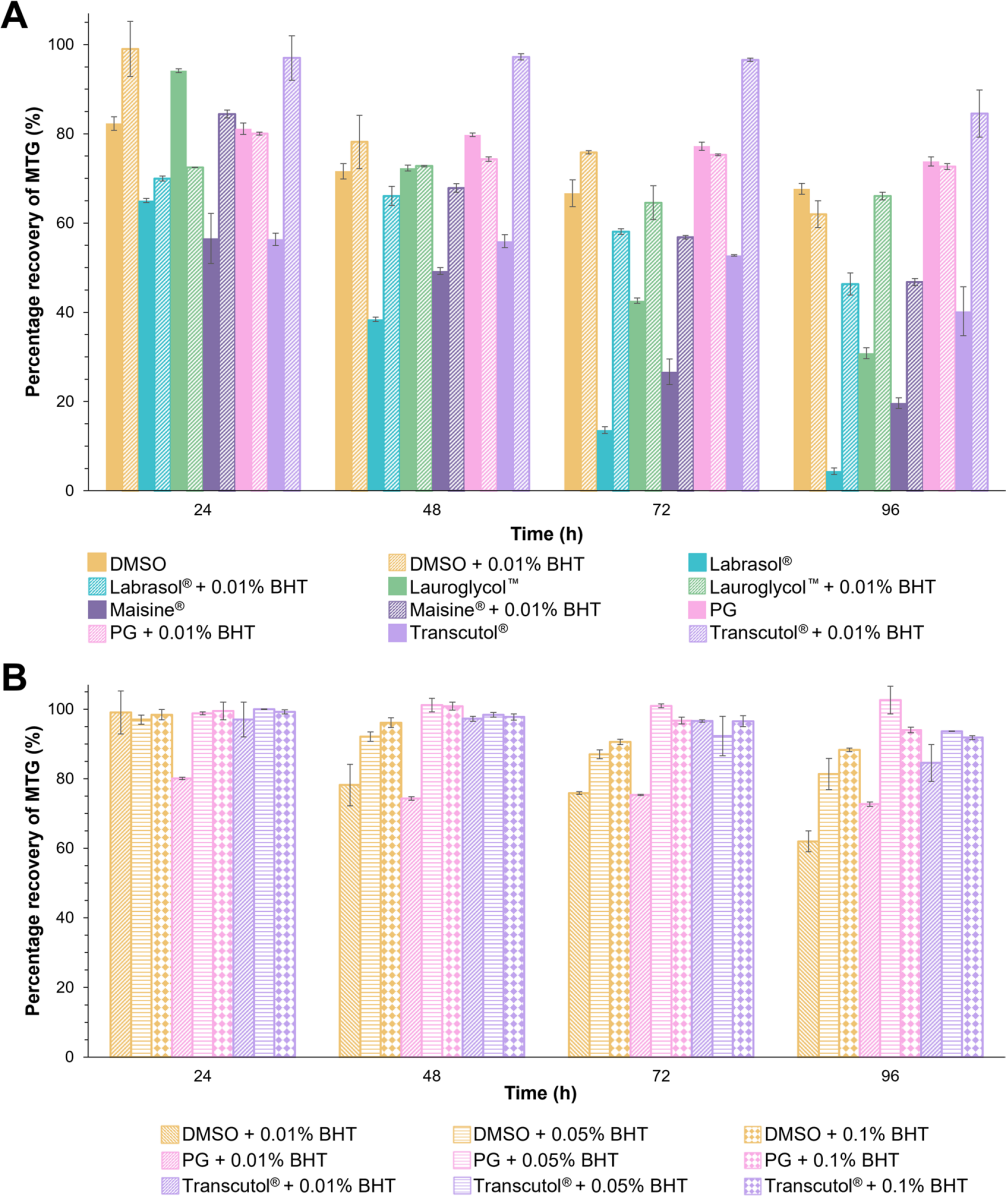
Stability studies of MTG (**A**) in solvents with and without 0.01%w/v of BHT and (**B**) in selected solvents with different BHT concentrations (*n* = 3, mean ± SD)

Figure 4A demonstrates the stability of MTG in the solvents used for the in vitro permeation studies. A decrease in the percentage recovery of MTG was generally observed across all solvents after 96 h. The recovery of MTG in PG was the highest (73.82 ± 1.05%) which was comparable to DMSO (67.68 ± 1.22%). The remaining of the solvents showed a drug recovery of < 50% (Transcutol^®^: 40.23 ± 5.46%; Lauroglycol™: 30.84 ± 1.21%; Maisine^®^: 19.65 ± 1.17% and Labrasol^®^: 4.39 ± 0.70%).

The addition of 0.01%w/v of BHT improved MTG recovery in solvents where initial recovery was below 50% (Fig. 4A) such as Transcutol^®^, Lauroglycol™ and Maisine^®^, effectively doubling the recovery rates. Notably, Labrasol^®^ exhibited a tenfold increase in MTG recovery, rising from 4.39 ± 0.70% to 46.34 ± 2.49%. In contrast, the inclusion of BHT in DMSO and PG did not yield any improvement, as MTG recovery in these solvents was already high even without the antioxidant. BHT, which is generally recognised as safe, was selected for use in solvent systems due to its potent antioxidant activity at low concentrations (< 0.1%) and its high solubility across a wide range of organic solvents [76]. While ascorbic acid also exhibits antioxidant properties, its hydrophilic nature renders it more suitable for aqueous formulations rather than solvent-based systems.

A further stability study for optimisation of BHT content was conducted in the selected solvents which demonstrated higher skin permeation including DMSO, PG and Transcutol^®^. An increase in the percentage recovery of MTG was observed (Fig. 4B) when the concentration of BHT was increased from 0.01%w/v to 0.1%w/v. The percentage recovery of MTG was generally above 80% for all solvents with at least 0.05%w/v of BHT. Hence, 0.05%w/v of BHT was selected for the gel formulations.

### Evaluation of transdermal delivery of MTG in gels

**Fig. 5:**
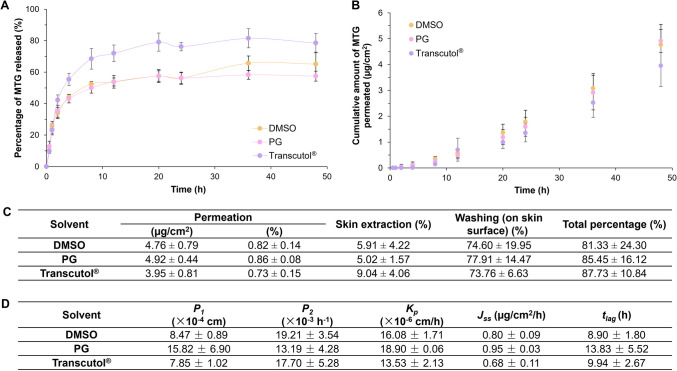
(**A**) In vitro drug release profiles and (**B**) skin permeation profiles of MTG from gels formulated with different solvents as well as (**C**) the relevant mass balance results and (**D**) skin permeation parameters (*n = *3, mean ± SD)

Based on the previous results, DMSO, PG and Transcutol^®^ were selected to be incorporated into the gel formulations. The drug content analysis demonstrated a good recovery of MTG from the gel formulations (DMSO: 108.66 ± 0.27%; PG: 103.66 ± 1.30%; Transcutol^®^: 92.70 ± 1.68%, *n = *3; mean ± SD) [77]. In vitro drug release studies (Fig. 5A) showed that all gels exhibited a steady release of MTG up to 2 h. Among the solvents, Transcutol^®^ achieved the highest drug release percentage (78.48 ± 6.17%), outperforming DMSO (65.07 ± 7.59%) and PG (57.45 ± 3.18%), which showed lower but comparable release profiles. Despite these differences in the initial release, all gels maintained a sustained drug release over a period of 48 h.

Despite the different drug release performance, the in vitro permeation profiles were almost similar for all gels (Fig. 5B). PG exhibited a slightly higher cumulative amount of MTG permeated (4.92 ± 0.44 μg/cm^2^) as compared to DMSO (4.76 ± 0.79 μg/cm^2^) and Transcutol^®^ (3.95 ± 0.81 μg/cm^2^). The mass balance results (Fig. 5C) indicated a total drug recovery of over 80%, which falls within the acceptable range of 80–120%. This outcome is likely attributed to the incorporation of BHT as an antioxidant. The findings underscore the significance of including an antioxidant in the formulation to enhance the stability of MTG and minimise drug loss due to degradation.

Further analysis of the skin permeation parameters (Fig. 5D) following the application of the gel formulations revealed that PG exhibited a higher skin partitioning effect, as indicated by its high *P*_*1*_ value, which was approximately twice that of DMSO and Transcutol^®^. This enhanced partitioning also contributed to the highest *K*_*p*_ observed for PG. In contrast, DMSO and Transcutol^®^ demonstrated higher *P*_*2*_ values, suggesting improved drug diffusion across the skin and supporting their roles in facilitating MTG permeation. Among all formulations, PG achieved the highest *J*_*ss*_ of 0.95 μg/cm^2^/h, exceeding that of the solvent-only systems. Also, the *t*_*lag*_ (9–14 h) observed across all gel formulations is shorter than the solvent-only systems but it is still considered relatively long likely due to the low drug loading.

## Conclusion

The present study focused on preserving MTG permeation to facilitate accurate quantification and a deeper understanding of its skin permeation mechanisms across various solvents. Our work demonstrated that the drug amorphicity and oxidation could be the main reasons for the drug degradation. In order to address this issue, the use of a minimal amount such as 0.01%w/v of ascorbic acid was found promising to preserve drug content for quantification without interfering drug permeation although the inclusion of additives in the receptor medium is not always ideal. With the aid of mathematical modelling, this study demonstrated that while skin diffusion serves as the primary mechanism of MTG permeation, skin partitioning emerges as a critical factor in enhancing its overall permeation. This is well reflected in the most effective solvents including DMSO, PG, and Transcutol^®^ enhanced the skin permeation of MTG which also contributed to a high skin flux. However, these solvents were associated with prolonged *t*_*lag*_ and drug degradation issues.

To ensure the successful development of MTG formulations such as gels, the incorporation of an antioxidant such as BHT, at a low concentration (0.05% w/v) and with good miscibility in the selected solvents, is crucial for minimising drug loss. Future research including solvent combinations and higher drug loadings should focus on addressing the skin flux and *t*_*lag*_ issues to inform further formulation strategies. Additionally, approaches aimed at stabilising MTG such as the development of a salt form may offer a viable solution to mitigate its chemical instability.

## Supplementary Information

Below is the link to the electronic supplementary material.

Supplementary file1 (DOCX 260 KB)

## Data Availability

The authors confirm that the data supporting the findings of this study are available within the article and its supplementary materials.
