# Supplementary material for: In vitro skin permeation of mitragynine: Optimisation of antioxidants for enhanced drug stability and formulation performance
Source: Drug Deliv Transl Res. 2025 Aug 16;15(11):4202–16. doi: 10.1007/s13346-025-01933-6 (PMC12507957; doi:10.1007/s13346-025-01933-6)
Supplement: Supplementary file 1 — Supplementary file1 (DOCX 260 KB) [file 13346_2025_1933_MOESM1_ESM.docx]

# Supplementary material:

***In vitro* skin permeation of mitragynine: Optimisation of antioxidants for enhanced drug stability and formulation performance**

Yee Shan Sim ^a^, Juzaili Azizi ^b^, Nelson Jeng-Yeou Chear ^b^, Siti Rafidah Yusof ^b^, Vikneswaran Murugaiyah ^b, c^, Su Yean Teh ^d^, Choon Fu Goh ^a^

^a^ Discipline of Pharmaceutical Technology, School of Pharmaceutical Sciences, Universiti Sains Malaysia, 11800 Minden, Penang, Malaysia

^b^ Centre for Drug Research, Universiti Sains Malaysia, 11800 Minden, Penang, Malaysia

^c^ Discipline of Pharmacology, School of Pharmaceutical Sciences, Universiti Sains Malaysia, 11800 Minden, Penang, Malaysia

^d^ School of Mathematical Sciences, Universiti Sains Malaysia, 11800 Minden, Penang, Malaysia


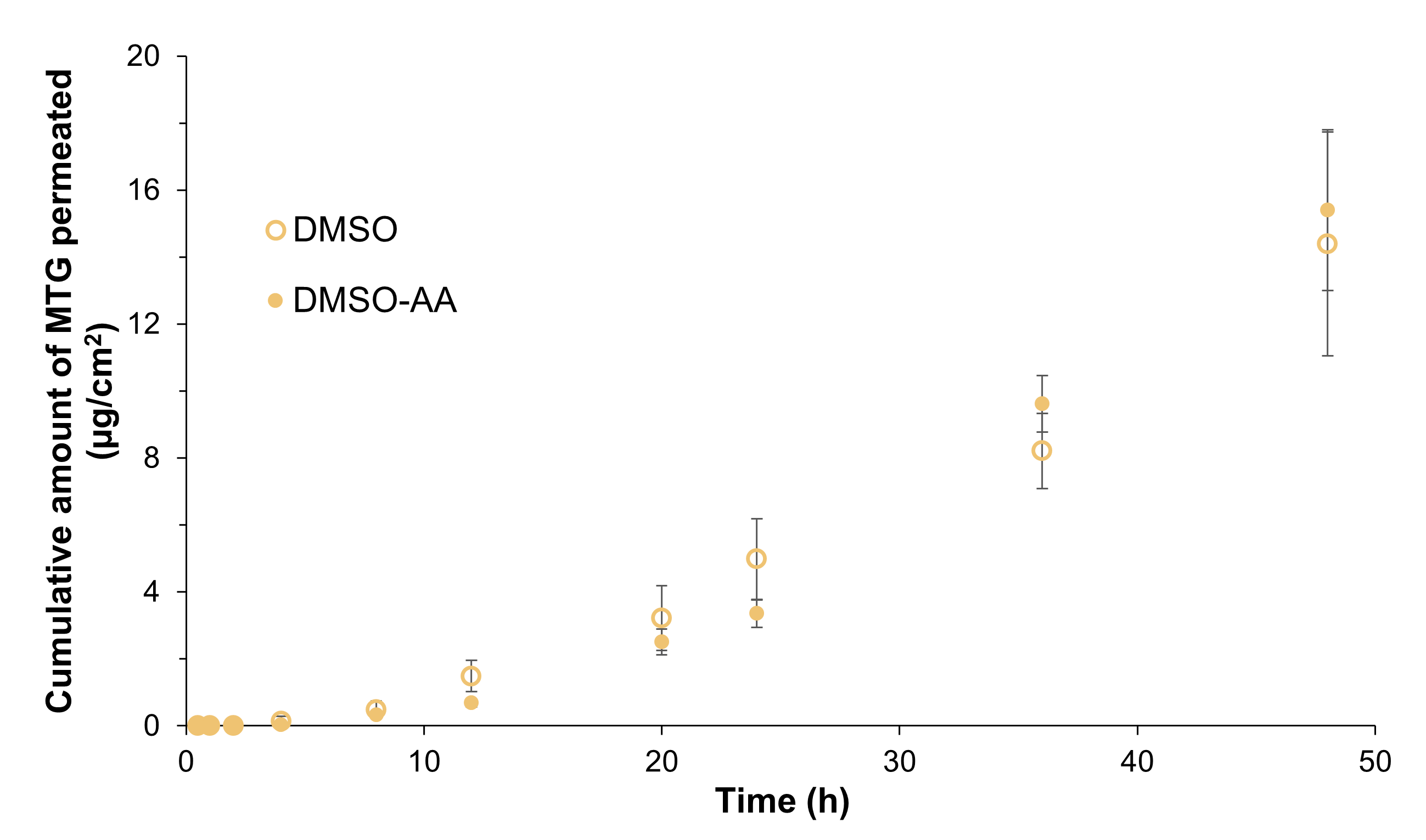


**Figure S1 Permeation profiles of 5%w/v of mitragynine in DMSO with and without 0.01%w/v ascorbic acid over 48 h (n = 3, mean ± SD)**


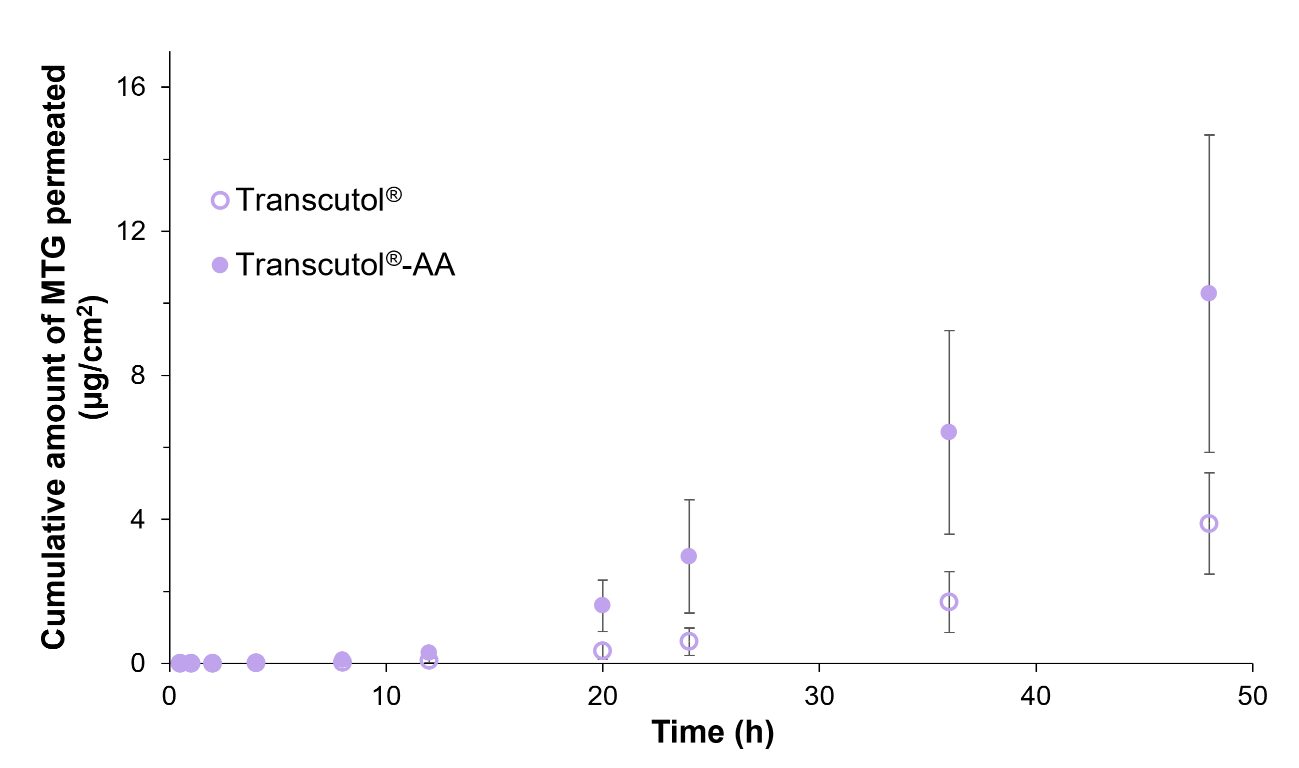


**Figure S2 Permeation profiles of 5%w/v of mitragynine in Transcutol^®^ with and without 0.01%w/v ascorbic acid over 48 h (n = 3, mean ± SD)**


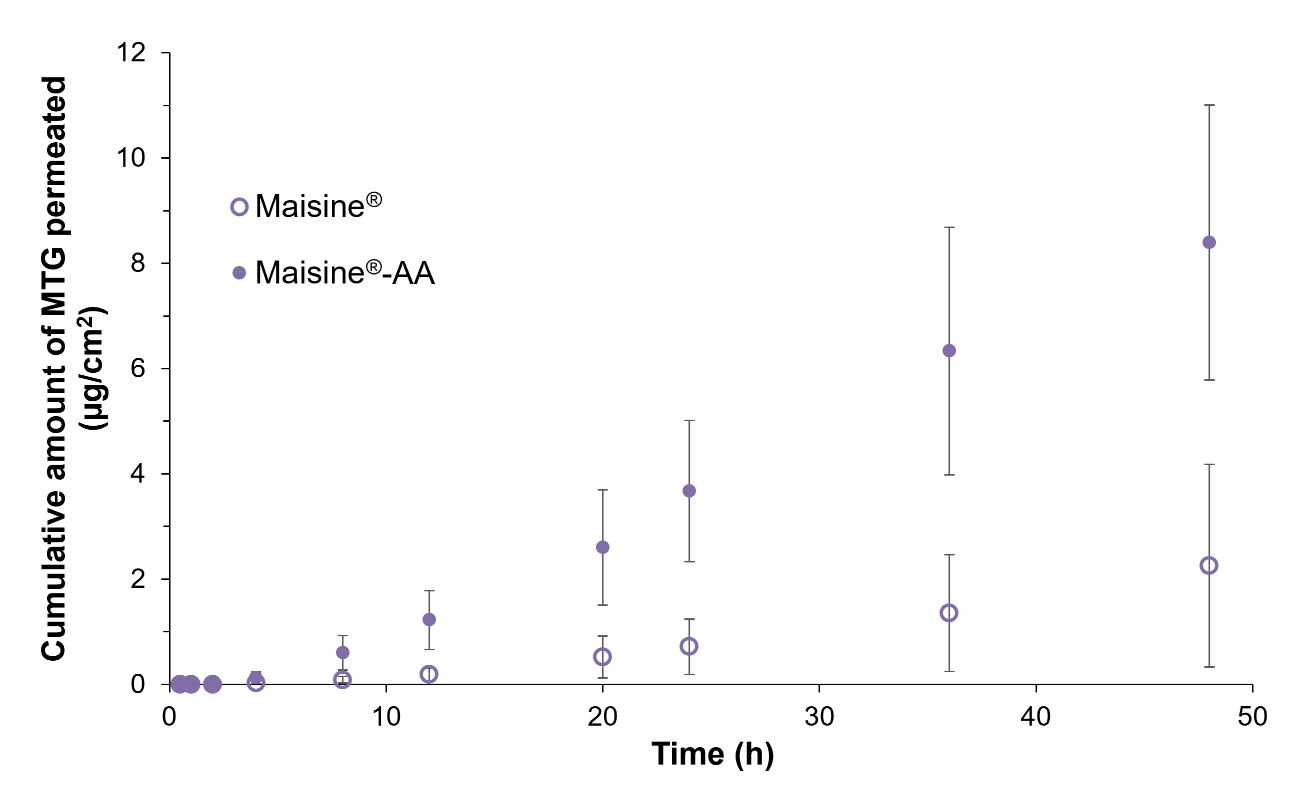


**Figure S3 Permeation profiles of 5%w/v of mitragynine in Maisine^®^ with and without 0.01%w/v ascorbic acid over 48 h (n = 3, mean ± SD)**

**
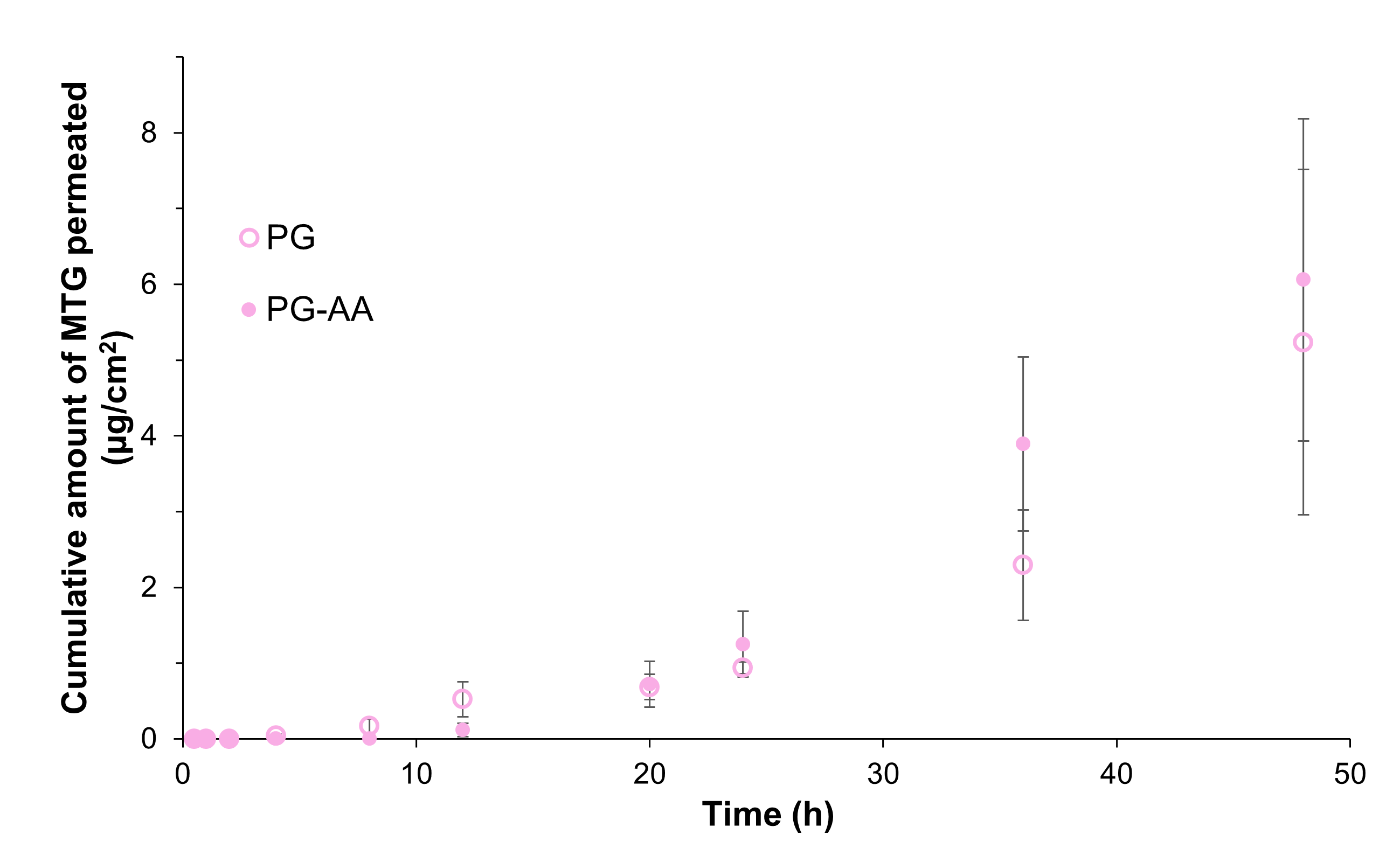
**

**Figure S4 Permeation profiles of 5%w/v of mitragynine in PG with and without 0.01%w/v ascorbic acid over 48 h (n = 3, mean ± SD)**


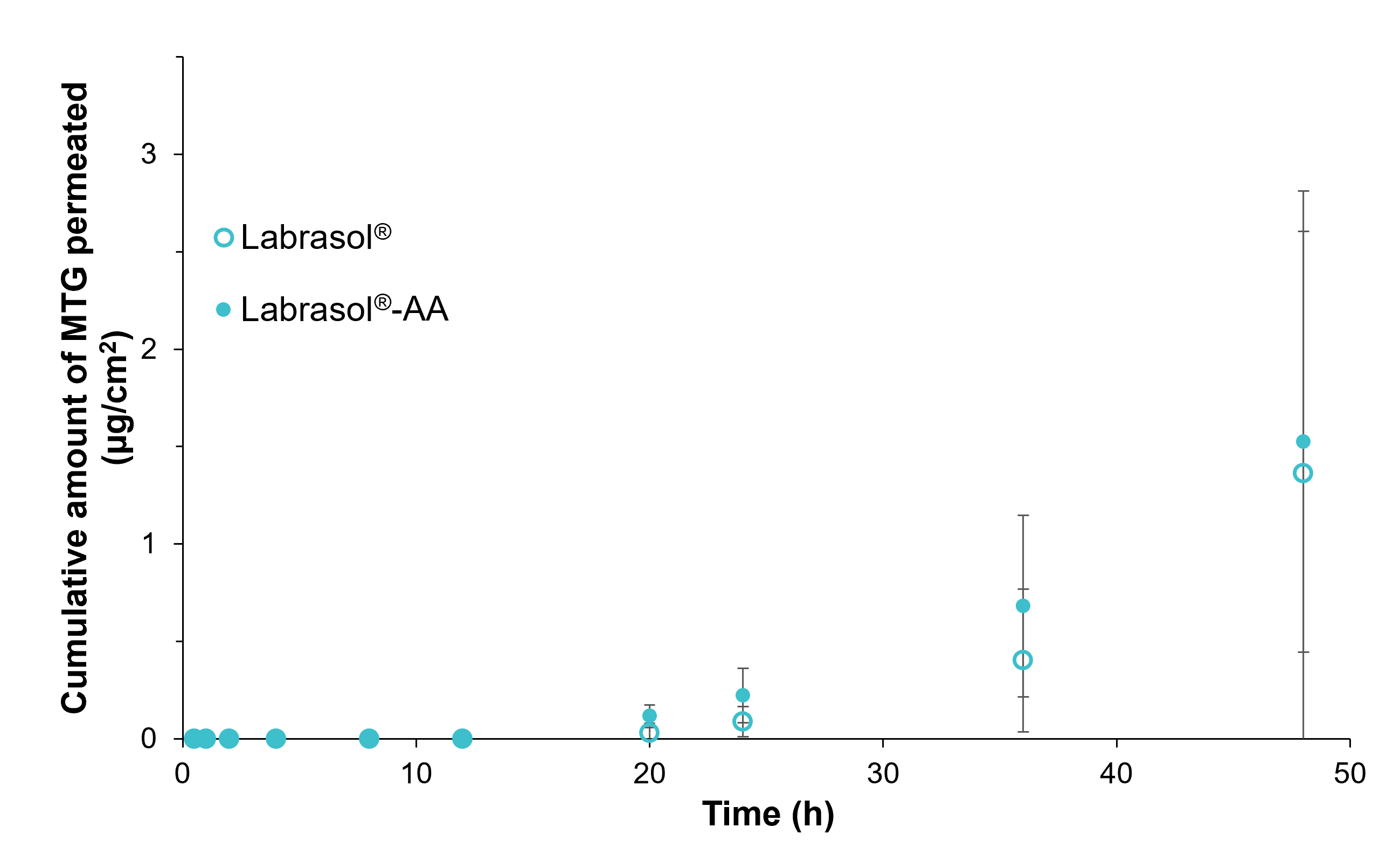


**Figure S5 Permeation profiles of 5%w/v of mitragynine in Labrasol^®^ with and without 0.01%w/v ascorbic acid over 48 h (n = 3, mean ± SD)**


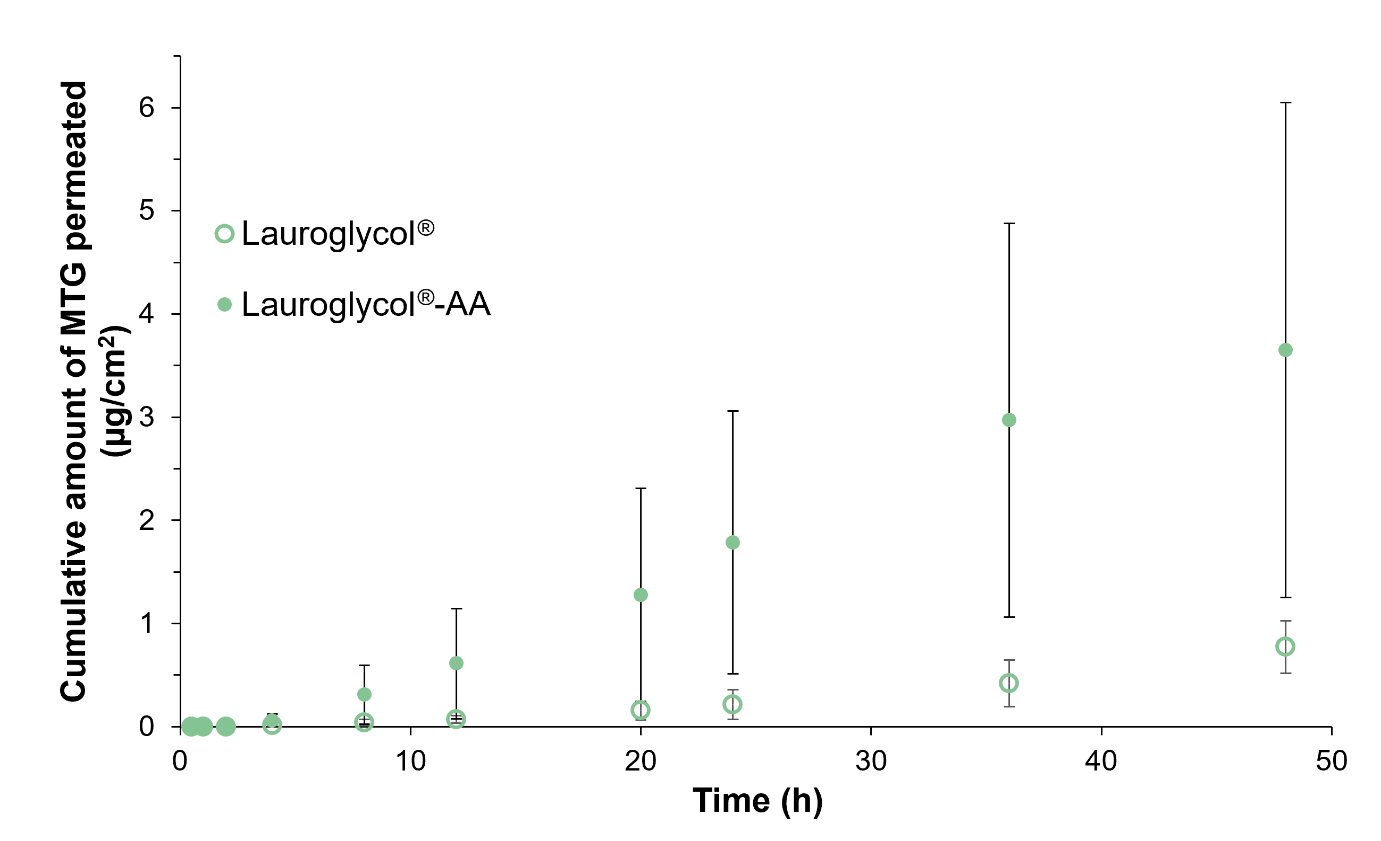


**Figure S6 Permeation profiles of 5%w/v of mitragynine in Lauroglycol^™^ with and without 0.01%w/v ascorbic acid over 48 h (n = 3, mean ± SD)**
